# In Situ Determination of Bisphenol A in Beverage Using a Molybdenum Selenide/Reduced Graphene Oxide Nanoparticle Composite Modified Glassy Carbon Electrode

**DOI:** 10.3390/s18051660

**Published:** 2018-05-22

**Authors:** Rongguang Shi, Jing Liang, Zongshan Zhao, Yi Liu, Aifeng Liu

**Affiliations:** 1 Key Laboratory for Environmental Factors Control of Agro-Product Quality Safety, Agro-Environmental Protection Institute, Ministry of Agriculture, Tianjin 300191, China; shirongguang@aepi.org.cn; 2 CAS Key Laboratory of Biobased Materials, Qingdao Institute of Bioenergy and Bioprocess Technology, Chinese Academy of Sciences, Qingdao 266101, China; liangjing@qibebt.ac.cn (J.L.); Liuaf@qibebt.ac.cn (A.L.); 3 School of Chemistry and Chemical Engineering, Yantai University, Yantai 264005, China

**Keywords:** electrochemical sensor, molybdenum selenide, reduced graphene oxide, bisphenol A, beverage

## Abstract

Due to the endocrine disturbing effects of bisphenol A (BPA) on organisms, rapid detection has become one of the most important techniques for monitoring its levels in the aqueous solutions associated with plastics and human beings. In this paper, a glassy carbon electrode (GCE) modified with molybdenum selenide/reduced graphene oxide (MoSe_2_/rGO) was fabricated for in situ determination of bisphenol A in several beverages. The surface area of the electrode dramatically increases due to the existence of ultra-thin nanosheets in a flower-like structure of MoSe_2_. Adding phosphotungstic acid in the electrolyte can significantly enhance the repeatability (RSD = 0.4%) and reproducibility (RSD = 2.2%) of the electrode. Under the optimized condition (pH = 6.5), the linear range of BPA was from 0.1 μM–100 μM and the detection limit was 0.015 μM (S/*N* = 3). When using the as-prepared electrode for analyzing BPA in beverage samples without any pretreatments, the recoveries ranged from 98–107%, and the concentrations were from below the detection limit to 1.7 μM, indicating its potential prospect for routine analysis of BPA.

## 1. Introduction

Bisphenol A (BPA), an endocrine-disrupting chemical (EDC) [1], has been of great concern as a result of its adverse physiological effects and frequent detections in various environmental mediums [2,3,4]. Since BPA has been widely used for the production of cups and packing materials [5,6,7], its migration from storage or packaging material to food or beverage [8] has been proposed as one of the important exposure pathways for human beings. The European Food Safety Authority (EFSA) has established a temporary Tolerable Daily Intake (t-TDI) of 4 μg/kg b.w./day for BPA [9]. Therefore, BPA usage in some infant food packages and bottles has been banned by legislation [10]. 

To monitor the environmental levels, behaviors and fate of BPA, many methods, such as capillary electrophoresis [11], gas chromatography coupled with mass spectrometry (GC-MS) [12], high performance liquid chromatography mass spectrometry (HPLC-MS) [13], enzyme linked immune sorbent assay (ELISA) [14], electrochemical methods [15,16], etc., have been developed. Combined with pretreatment procedures of solid-phase extraction [17], liquid-liquid extraction [18] and immunoaffinity chromatography [19], GC-MS, HPLC-MS and ELISA can be used for selectively and sensitively determining BPA in complex matrices [20,21]. However, the complementary pretreatments and expensive equipment have restricted their application. 

Electrochemical methods are some of the preferable techniques for routine analysis of some chemical compounds owing to the inherent advantages of the low cost of the instrument, ease of preparation, high sensitivity, simplicity for operators and in situ monitoring [22]. To improve the response signals of substances, a series of modified electrodes has been developed. For example, electrodes based on graphene nanomaterials present excellent electric conductivity, as well as chemical and thermal stability [23,24]. Electrodes modified with molybdenum-selenide (MoSe_2_) show very good sensitivity as a result of increasing the surface active sites [25,26]. 

MoSe_2_/graphene composites are proposed as useful materials for fabricating electrochemical sensors and biosensors due to their excellent physical and chemical properties of large surface and better conductivity [27]. In this paper, a novel electrochemical sensor based on molybdenum selenide/reduced graphene oxide (MoSe_2_/rGO) nanocomposites has been developed for in situ determination of BPA in beverages. The performance of the electrode has been evaluated by analyzing BPA in some commercial beverages without any pretreatments or purification.

## 2. Experimental

### 2.1. Reagents and Materials

BPA, phosphotungstic acid (H_3_[PW_12_O_4_], PTA), graphite powders, Se powder, sodium molybdate, sodium hydroxide (NaOH), potassium permanganate (KMnO_4_), sulfuric acid (H_2_SO_4_, 98%), sodium nitrate (NaNO_3_) and phosphoric acid (H_3_PO_4_) were purchased from Sinopharm Group Chemical Regent Co. Ltd. (Shanghai, China). BPA was dissolved in ethanol and kept at 4 °C. PTA (1 mM) and phosphate buffer solution (PBS, 1 M, pH = 6.5) were used as reduced and supporting electrolytes. All chemicals used were at least analytical grade. Ultrapure water (18.2 MΩ·cm, obtained by Milli-Q Water Purification System, Billerica, MA, USA) was used for preparing all buffers and standard solutions. 

### 2.2. Instruments

All cyclic voltammetry (CV) measurements were performed using a CHI660E electrochemical workstation (Chenhua Instruments, Shanghai, China) with a conventional three-electrode system. A glassy carbon electrode (GCE) was used as the working electrode, with an Ag/AgCl electrode and a platinum wire electrode as the reference electrode and counter-electrode, respectively. Measurements of pH were carried out by a Mettler Toledo Delta 320 pH meter (Shanghai, China). The morphologies of materials and electrodes were obtained using transmission electron microscopy (TEM) (Hitachi H-7650, Hitachi High-Technologies Corporation, Japan). The X-ray diffraction (XRD) of the samples was carried out using Bruker XRD (Bruker D8 advance Diffracto meter, Bruker Germany).

### 2.3. Preparation of MoSe_2_/rGO Composite

The MoSe_2_ nanoparticles (NPs) were synthesized by the hydrothermal method [28]. Briefly, Se solution (5 mL, 0.4 M in hydrazine hydrate) was added dropwise to sodium molybdate solution (50 mL, 0.02 M). The mixture was transferred into a 100 mL Teflon-lined autoclave and heated at 180 °C for 48 h. The black MoSe_2_ nanoparticles were obtained after washing with distilled water and drying in a vacuum. The graphite oxide (GO) was prepared according to previous reports [29].

The preparation of the MoSe_2_/rGO was completed as below. The synthesized MoSe_2_ nanoparticles (0.5 mL, 1 mg/mL in water) were mixed with GO (10 mL, 1 mg/mL in water) and stirred for 2 h. MoSe_2_/GO (5 μL) was cast onto the pre-polished and cleaned GCE surface and dried naturally. Then, MoSe_2_/rGO nanoparticle films were prepared on the GCE surface by the electrochemical reduction process. The electrolytes were phosphate buffer solutions. The reduced potential was −1.2 V, and the time was 120 s, respectively. A similar procedure was also used to fabricate modified electrodes of MoSe_2_ NPs/GCE and rGO/GCE.

### 2.4. Analysis of Real Sample

Coffee, soybean milk powder, prepared juice, liquid milk and orange juice samples obtained from a local supermarket were used for evaluating the performance of the prepared electrode. Liquid samples of liquid milk and orange juice were directly mixed with PBS solution for analysis without any pretreatment. For coffee, soybean milk powder and prepared juice, certain amounts (2.5 g) of these solid samples were firstly mixed with 25 mL ultrapure water in plastic bottle and then heated to 100 °C for 1 h. Finally, the obtained solutions were cooled to room temperature (25 °C) for further analysis after spiking with BPA standard solutions. 

## 3. Results and Discussion

### 3.1. Characterization of Electrode Materials

**Figure 1:**
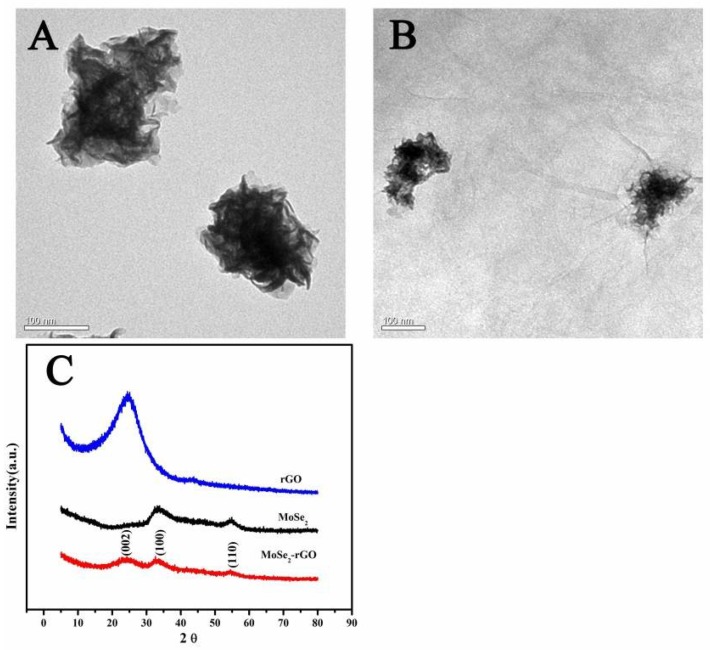
Characterization of the prepared electrode materials. (**A**) TEM images of MoSe_2_ nanoparticles; (**B**) TEM images of the MoSe_2_/rGO composite; and (**C**) XRD spectrum of the rGO, MoSe_2_ and MoSe_2_/rGO composite.

The TEM images showed that MoSe_2_ nanoparticles possess flower-like structures being composed of ultra-thin nanosheets, and their diameters were about 200 nm (Figure 1A). In the MoSe_2_/GO composites, it was obvious that the layered MoSe_2_ nanosheets have been well wrapped in the thin graphene film (Figure 1B). The characteristic diffraction peaks were indexed to (002), (100) and (110) planes from the XRD pattern of MoSe_2_/rGO composites corresponding to that of rGO and monoclinic MoSe_2_ nanoparticles (JCPDS: 77-1715), indicating the successful combination of MoSe_2_ and rGO.

### 3.2. Enhanced Electrochemical Properties of MoSe_2_/rGO/GCE

Cyclic voltammogram (CV) were employed to study the electrochemical properties of the bare GCE and modified electrode (Figure S1) in 5 mM K_3_[Fe(CN)_6_]^3−/4−^ solution (containing 0.1 M KCl). The electrochemical response of the ferricyanide probe was a reversible process. At a scan rate of 100 mV/s, the pairs of redox peak currents of bare GCE, rGO/GCE and MoSe_2_/rGO/GCE were 6.3 µA, 15.6 µA and 39.7 µA, respectively. The higher redox current responses of MoSe_2_/rGO/GCE than the bare GCE and rGO/GCE could be attributed to its larger surface area due to the introduction of the hierarchy structures of MoSe_2_ nanoparticles. The better electron transfer resulting from rGO also led to the smaller peak-to-peak potential separation (ΔEp) value of the MoSe_2_/rGO/GCE (0.089 V).

**Figure 2:**
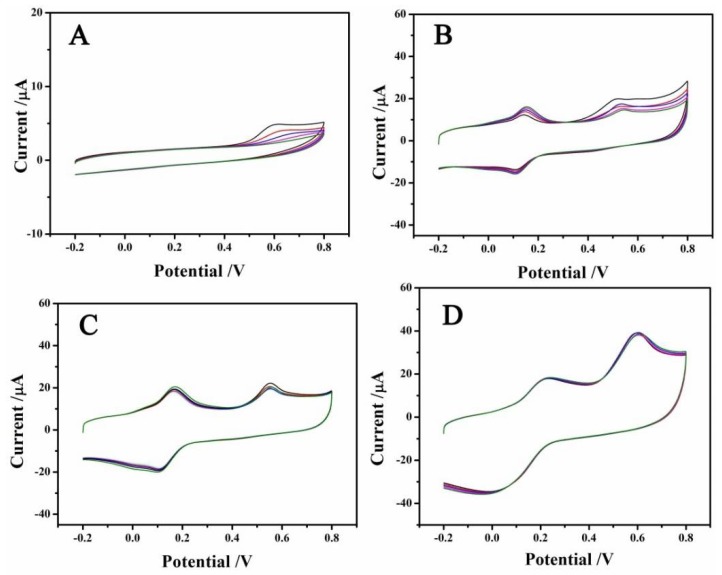
CVs of different electrodes for detecting BPA (0.01 mM in PBS solution, pH = 6.5) at a scan rate of 100 mV/s. (**A**) bare GCE; (**B**) rGO/GCE in the absence of phosphotungstic acid (PTA); (**C**) rGO/GCE in the presence of PTA; (**D**) MoSe_2_/rGO/GCE in the presence of PTA.

PTA is proposed as a recommend substance for improving the sensitivity, stability and repeatability of the electrodes [30]. Here, its contribution to the oxidation peak of BPA has also been evaluated. In the detection system, the electrolyte was composed of 1 mL PTA (0.1 mM), 1 mL BPA (0.01 mM), 1 mL PBS (1 M, pH = 6.5) and 7 mL H_2_O. Such a low level of PTA had no effect on the pH value of the electrolyte adjusted by PBS. In the absence of PTA, the cyclic voltammetric was carried out in the potential range of −0.2 V–0.8 V using GCE as the working electrode. A single smaller oxidation peak (2.9 µA) appeared at about 0.6 V in the beginning due to the oxidation potential of the irreversible phenolic hydroxyl of BPA (Figure 2A) [31], and it disappeared completely after the fourth run. The reversible redox peaks around 0–0.2 V (Figure 2B), which did not interfere with determination peak of BPA, were due to the conversion between hydroxyl and carboxyl groups on the graphene [32]. The attenuated oxidation currents of BPA at around 0.6 V were observed for rGO/GCE after several runs, though the oxidation current was much higher (16.8 µA) at the first scan (Figure 2B). The attenuated signals can be largely attributed to the serious surface deactivation of the electrodes for BPA. In the presence of PTA (0.1 mM), the CV signals of both rGO/GCE (Figure 2C) and MoSe_2_/rGO/GCE (Figure 2D) presented much better stability. After the fifth run, the oxidation currents still reached up to 88% (RSD = 5.48%, *n* = 5) and 98.7% (RSD = 0.43%, *n* = 5) of the initial peak current for rGO/GCE and MoSe_2_/rGO/GCE, respectively. The enhancement of the stability of the oxidation signals of BPA should be attributed to the anti-fouling capacity of PTA, which can oxidize the phenoxyl radical to the carbocation [32]. Additionally, the better stability (RSD = 0.43%, *n* = 5) and sensitivity (26.5 μA) of MoSe_2_/rGO/GCE compared to rGO/GCE could be ascribed to the larger surface of MoSe_2_ nanoparticles, which can increase the active sites and facilitate the electron exchange.

### 3.3. Reaction Mechanism of BPA on the Surface of MoSe_2_/rGO/GCE

**Figure 3:**
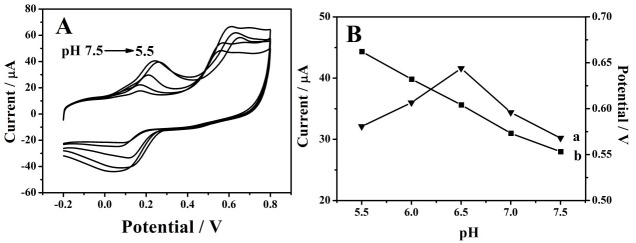
(**A**) CVs of BPA (0.1 µM) using MoSe_2_/rGO/GCE with different pHs (from 5.5–7.5); (**B**) variation of peak current (a) and peak potential (b) with different pHs. The scan rate was 100 mV/s, and the electrolyte was 0.1 M PBS.

It has been reported that the reaction of BPA on the surface of MoSe_2_/rGO/GCE is proton-dependent (0.1 mM), and the oxidation potential would change with pH variations of the electrolyte [22]. As shown in Figure 3, the highest oxidation current toward the oxidation of BPA on the surface of MoSe_2_/rGO/GCE appeared at a pH of 6.5. Additionally, the oxidation potentials (*E_pa_*) of BPA were inversely associated with the pH values (Figure 3B). The regression equation could be expressed as *E_pa_* (V) = −0.0554 pH + 0.9641 (*R*^2^ = 0.994). The slope of 0.0554 V/pH was close to the theoretical value of 0.0576 V/pH [22], indicating that the electron transfer was accompanied by an equal number of proton consumption in the electrode reaction of BPA. 

**Figure 4:**
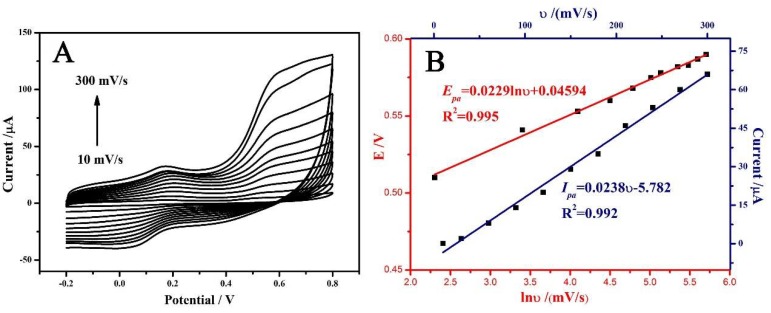
(**A**) CVs of BPA at MoSe_2_/rGO/GCE in 0.1 M PBS (pH = 6.5) at different scan rates; (**B**) the relationship of peak currents (*I_pa_*) with the scan rates (υ) and peak potentials (*E_pa_*) with the scan rates.

The relationship of currents and the scan rate was investigated to understand the reaction kinetics of BPA on the surface of MoSe_2_/rGO/GCE. The peak currents (*I_pa_*) increased linearly with the scan rates (υ) in the range of 10–300 mV/s (Figure 4A), indicating that the reaction should be an adsorption-controlled process (Figure 4B) [33]. Additionally, there is a linear relationship between the oxidation peak potential (*E_pa_*) and the nature logarithmic scan rate (lnυ) (Figure 4B). The relationship can be expressed using the following regression equation: *E_pa_* (V) = 0.0229 lnυ + 0.4594 (*R*^2^ = 0.995). According to the Laviron equation, *E_pa_* of an irreversible electrode process can be defined by the following equation:

$$Epa=E^{0}+\left(\frac{RT}{\alpha nF}\right)\mathrm{In}\left(\frac{RT\kappa^{0}}{\alpha nF}\right)+\left(\frac{RT}{\alpha nF}\right)\mathrm{In}\upsilon$$

where *α* is the transfer coefficient, *k*^0^ is the rate constant of the electrochemical reaction, *n* is the number of electrons transferred, *υ* is the scan rate and *E*^0^ is the formal potential. Other symbols have their usual meanings (*R* = 8.314 J mol^−1^ K^−1^, *T* = 298 K, *F* = 96,480 C mol^−1^). Based on the slope of 0.0229, the calculated *αn* (*α* = 0.5) was 1.1213, indicating that the electron transfer number (*n*) for BPA oxidation was around two.

### 3.4. Interference Analysis

Under the optimum conditions (pH 6.5), the influence of the coexisting ions and BPA analogs for determining BPA (10.0 μM) by MoSe_2_/rGO/GCE has been tested, and the results are shown in Table S1. The inorganic substances of KCl, NaCl, CaCl_2_, BaCl_2_, CuCl_2_, FeCl_3_ and NaOH (1 mM) presented little effect, with the peak current suppression or enhancement less than 2.71%. More interestingly, it was also found that there was no obvious effect on the peak current variations of BPA oxidation when the concentration was 10-times BPA for its analogs of phenol, bisphenol S, bisphenol F and bisphenol B. The results suggested that the MoSe_2_/rGO/GCE possesses good selectivity and anti-interference ability towards BPA oxidation. 

### 3.5. Analytical Performance

**Figure 5:**
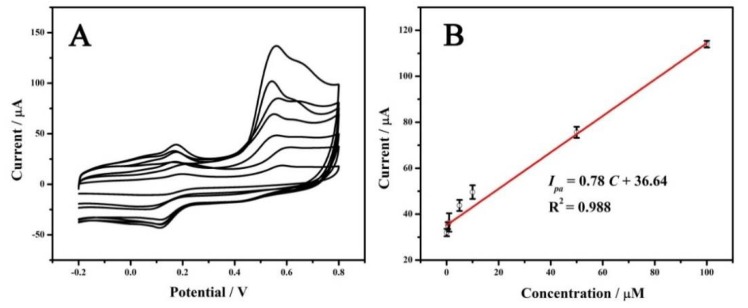
(**A**) CVs of MoSe_2_/rGO/GCE in 1 M PBS (pH 6.5) containing different concentrations of BPA (from the bottom to top: 0.1, 0.5, 1, 5, 10, 50, 100 µM); (**B**) the calibration line of the response *I_pa_* vs. C_BPA_.

Under the optimized conditions, CVs of BPA with different concentrations were recorded. As expected, the anodic peak current of BPA (*I_pa_*) at MoSe_2_-rGO/GCE gradually increased, accompanied by BPA levels increasing. The *I_pa_* of the BPA was the average value determined by five measurements. As no anodic peak appeared in the absence of BPA, no baseline correction was performed in our experiment. Ultimately, a linear regression equation *I_pa_* (µA) = 0.78 C (µM) + 36.64 (*R*^2^ = 0.988) was obtained when BPA concentrations ranged from 0.1 µM to 100 µM (Figure 5). The detection limit (DL) (S/*N* = 3) was 0.015 µM. Compared with other modified electrodes for BPA analysis detection limits (DLs > 0.04 µM) [34,35,36,37,38], the MoSe_2_/rGO/GCE electrode presented comparable or even better sensitivity, except for the Ni_2_Al-layered double hydroxide/GCE electrode [39] and the AuNPs/MoSe_2_/GCE electrode [40]. 

MoSe_2_/rGO/GCE also presented good stability, repeatability and reproducibility when analyzing dissolved BPA. After storing in refrigerator at 4 °C for two weeks, the response of MoSe_2_/rGO/GCE only reduced by less than 10%. The RSD of the response signals of individual MoSe_2_/rGO/GCE towards BPA oxidation (0.43%, *n* = 5) was much lower than some modified electrodes (Table 1). RSD obtained from three parallel prepared MoSe_2_/rGO/GCE was only 2.2%, still at a lower level for those modified electrodes determining BPA (Table 1).

### 3.6. Samples Analysis

Before adding BPA into the solutions containing liquid milk, orange juice, coffee, soybean milk and prepared juice, BPA in almost all these solutions was below the detection limit except that in the orange juice with a detectable BPA concentration of 1.7 µM (Table 2). The detected BPA was further identified and quantified by using HPLC-LTQ-MS (Figure S2). Based on the identification of the standards, BPA was observed at a retention time of 7.8 min with the specific ions at *m*/*z* of 227 ([M-H]^−^), 211.9 ([M-H-CH_3_]^−^) and 182.9 ([M-H-C_2_H_4_O]^−^) [41], and its concentration was about 1.8 µM. After spiking at three levels (10, 15 and 20 µM), BPA could be detected in all the solutions (Table 2). The recoveries ranged from 98% to 107%, and the calculated RSDs were all lower than 3%. These results indicated that the MoSe_2_/rGO/GCE sensor could be a potential strategy for routine analysis of BPA, especially in some liquid samples [41].

## 4. Conclusions

In summary, a simple electrochemical sensor based on MoSe_2_/rGO-modified GCE has been successfully fabricated for in situ determination of BPA in aqueous samples. Combined with the good conductivity of rGO and the positive catalysis of MoSe_2_, the MoSe_2_/rGO composites provided a favorable sensitivity for analyzing BPA. The prepared MoSe_2_/rGO/GCE sensor displayed a wide linear range, low detection limit, good stability, repeatability, reproducibility and high recoveries. The good performance for in situ determination of BPA in real samples indicated that the MoSe_2_/rGO/GCE sensor could be a potential strategy for routine analysis of BPA.

## Figures and Tables

**Table 1:** Comparison of the MoSe_2_/rGO modified electrode for BPA determination with other modified electrodes (C_BPA_ = 100 μM).

Modified Electrode	Linear Range (µM)	Detection Limit (µM)	Repeatability (RSD)	Reproducibility (RSD)	Medium	Reference
Magnetic non-imprinted NPs/CPE	0.6–100	0.1	*n* = 7, 1.4%	4.3%	water	[34]
polypyrrole/graphene quantum dots/GCE	0.1-50	0.04	2.2%	-	water	[23]
AuNPs/MoS_2_/GCE	0.05–100	0.005	*n* = 10, 2.35%	4.72%	rubber, water	[40]
β-cyclodextrin (β-CD)-ionic liquid/CPE	0.1–11	0.083	*n* = 6, 2.35%	5.09%	water, food package	[35]
Pt/graphene-CNTs	0.06–10, 10–80	0.042	*n* = 5, 5.3%	-	thermal printing papers	[36]
AuNPs/tacked graphene nanofibers/GCE	0.08–250	0.035	*n* = 10, 2.55%	4.46%	bottles	[37]
Ni_2_Al layered double hydroxide/GCE	0.02–1.51	0.0068	*n* = 5, 4.3%	-	milk	[39]
Hydroxylated-MWCNT/GCE	1–24	0.81	*n* = 11, 1.81%	-	water, bottles	[38]
MoSe_2_/rGO	0.1–100	0.012	*n* = 5, 0.429%	2.2%	beverages	This work

**Table 2:** Determination results of BPA in real samples.

Sample	Added (µM)	Found (μM, *n* = 5)	RSD (%, *n* = 5)	Recovery (%)
Liquid milk	0	<LOD		
	10	10.08 ± 0.243	2.40	100.8
	15	15.09 ± 0.067	0.45	100.6
	20	20.69 ± 0.361	1.18	103.5
Orange juice	0	1.7		
	10	10.16 ± 0.102	1.00	101.6
	15	15.66 ± 0.523	3.34	104.4
	20	20.73 ± 0.264	1.27	103.7
Coffee	0	<LOD		
	10	10.30 ± 0.282	2.75	103.0
	15	15.21 ± 0.212	1.39	101.4
	20	20.98 ± 0.552	2.62	104.9
Soybean milk	0	<LOD		
	10	9.88 ± 0.145	1.47	98.8
	15	15.41 ± 0.260	1.68	102.7
	20	20.47 ± 0.250	1.25	102.4
Prepared juice	0	<LOD		
	10	10.64 ± 0.306	2.87	106.4
	15	14.95 ± 0.342	2.29	99.8
	20	20.57 ± 0.291	1.42	102.9

## Supplementary Materials

The following are available online at http://www.mdpi.com/1424-8220/18/5/1660/s1, Table S1: Analytical results of BPA in the presence of interfering substances, Figure S1: CVs of (a) bare electrode, (b) rGO/GCE and (c) MoSe_2_/rGO/GCE in 5 mM [Fe(CN)6]^3−/4−^ containing 0.1 M KCl at a scan rate of 100 mV/s, Figure S2: Confirmation of bisphenol A in orange juice by HPLC-LTQ-MS.
