# Supplementary material for: In Situ Determination of Bisphenol A in Beverage Using a Molybdenum Selenide/Reduced Graphene Oxide Nanoparticle Composite Modified Glassy Carbon Electrode
Source: Sensors (Basel). 2018 May 22;18(5):1660. doi: 10.3390/s18051660 (PMC5982353; doi:10.3390/s18051660)
Supplement: Supplementary file 1 [file sensors-18-01660-s001.pdf]

Supporting Information

In Situ Determination of Bisphenol A in Beverage Using a Molybdenum Selenide/Reduced Graphene Oxide Nanoparticle Composite Modified Glassy Carbon Electrode

Rongguang Shi [1], Jing Liang [2], Zongshan Zhao [2,*], Yi Liu [3,*] and Aifeng Liu [2]

[1] Key Laboratory for Environmental Factors Control of Agro-Product Quality Safety, Agro-Environmental Protection Institute, Ministry of Agriculture, Tianjin 300191, China; shirongguang@aepi.org.cn

[2] CAS Key Laboratory of Biobased Materials, Qingdao Institute of Bioenergy and Bioprocess Technology, Chinese Academy of Sciences, Qingdao 266101, China; liangjing@qibebt.ac.cn (J.L.); Liuaf@qibebt.ac.cn (A.L.)

[3] School of Chemistry and Chemical Engineering, Yantai University, Yantai 264005, China

[*] Correspondence: zhaozs@qibebt.ac.cn (Z.Z.); liuyi@ytu.edu.cn (Y.L.); Tel.: +86-532-80662709 (Z.Z.)

Table S1. Analytical results of BPA in the presence of interfering substances.

Interference	Concentration (mM)	Signal change (%)	RSD[a] (%)
KCl	1.0	-1.23	1.23
NaCl	1.0	+1.26	1.42
$CaCl_2$	1.0	+1.37	2.29
$BaCl_2$	1.0	-2.32	1.61
$CuCl_2$	1.0	+2.71	2.64
$FeCl_3$	1.0	+0.32	1.37
NaOH	1.0	+0.94	2.35
phenol	0.1	-1.89	1.14
bisphenol S	0.1	+2.35	2.47
bispehnol F	0.1	+0.43	1.38
bisphenol B	0.1	+2.33	3.23

[a] The results are the arithmetic means from 3 parallel determination values. The concentrations of BPA, inorganic salts and BPA analogs are 10 μM, 1 mM and 100 μM, respectively. The electrolyte is PBS and the pH is 6.5.

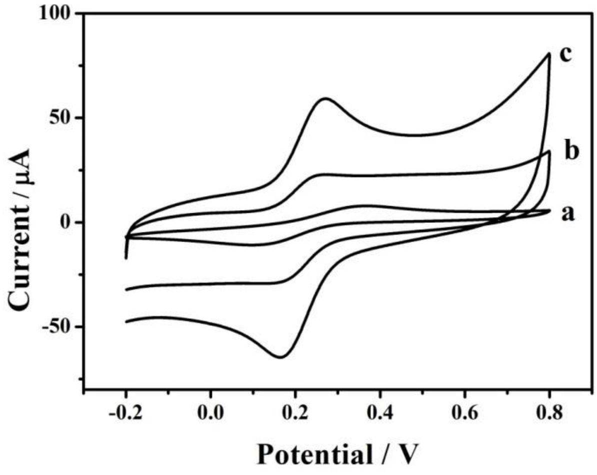

Figure S1. CVs of (a) bare electrode, (b) rGO/GCE and (c) $MoSe_2$/rGO/GCE in 5 mM $[Fe(CN)6]^{3-/4-}$ containing 0.1 M KCl at a scan rate of 100 mV/s.

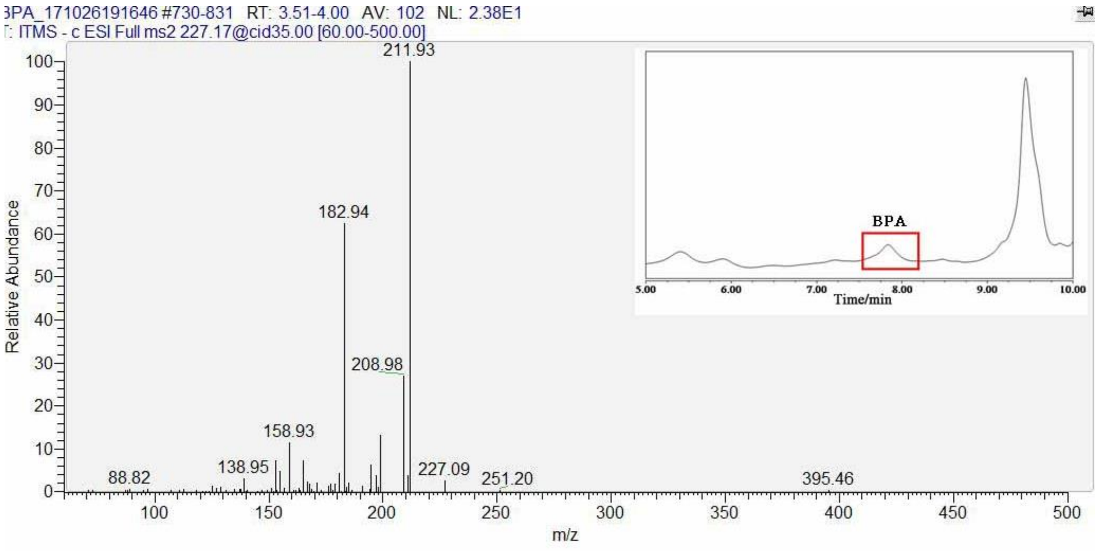

Figure S2. Confirmation of bisphenol A in orange juice by HPLC-LTQ-MS.
